# Delayed Rupture of a Cortical Traumatic Intracranial Aneurysm

**DOI:** 10.7759/cureus.3643

**Published:** 2018-11-27

**Authors:** Evan M Krueger, Ryan Trombly, Gina Guglielmi, Hamad Farhat

**Affiliations:** 1 Neurosurgery, Advocate Health Care, Downers Grove, USA; 2 Neurosurgery, Advocate Bromenn Medical Center, Chicago, USA; 3 Neurosurgery, Advocate Christ Medical Center, Oak Lawn, USA

**Keywords:** traumatic intracranial aneurysm, delayed aneurysm rupture, atypical cerebral bleeding, aneurysm clipping

## Abstract

Traumatic intracranial aneurysms are rare lesions that occur after blunt or primarily penetrating mechanisms. These are extremely fragile vessel injuries associated with significant morbidity and mortality, especially after rupture. Disease natural history, surveillance strategies, and management are based on small case series. Here we present a case of a 29-year-old male with a large epidural hematoma after blunt trauma, who underwent emergent surgical intervention. Three months postoperatively, he presented with unusual cerebral bleeding. Clinical suspicion prompted a conventional angiogram, which diagnosed a ruptured cortical traumatic intracranial aneurysm. The patient was urgently treated by surgical clipping with a good outcome.

## Introduction

Traumatic intracranial aneurysms (TICA) are uncommon lesions traditionally associated with significant morbidity and mortality. They can arise indirectly from the stretching forces in blunt injury, or more commonly from direct injury from penetrating objects, iatrogenic sources, or secondary traumatic injury from adjacent anatomic structures such as the falx cerebri, clinoid process, or basilar skull. TICAs can be further classified histologically as true, false or pseudo, mixed, and dissecting. True TICAs result after an injury to the intima and media, with the adventitia remaining intact and forming the aneurysm wall [1]. Comparatively, traditional intracranial aneurysm walls consist of the intima and adventitia. False or pseudo-TICAs result from a full-layer arterial rupture with either a resolving hematoma or the surrounding structures creating a resultant cavity that forms the aneurysm wall. Mixed TICAs are defined as true TICAs that subsequently rupture and form pseudo-TICAs [1]. Lastly, dissecting TICAs result in false lumen formation between the intima and superficial layers. The distinguishing angiographic features of TICAs include location not at a typical branching point, an irregular caliber of the parent vessel or an aneurysm dome, poorly defined neck, and delayed filling and emptying [2].

TICAs constitute 0.23% to 0.64% of all aneurysms, although their true incidence is probably under-diagnosed since most are asymptomatic until rupture [3-4]. The most common subtypes are false [1]. They occur more commonly in the pediatric population and are typically found in the distal anterior cerebral artery and internal carotid artery [1,3-4].

These lesions are complex and difficult to manage, largely due to the unknown natural history and anatomic fragility. Unfortunately, most patients present with hemorrhage, which is subsequently associated with a higher mortality [3,5]. The mean time from trauma to presentation ranges widely from 17 to 230 days, which raises surveillance concerns [1-5]. Surgical mortality has been reported as 24%. Historically, untreated TICA mortality rates approached 50% [2]. Contemporary literature shows a Glasgow Outcome Score (GOS) of 5 in treated TICAs between 41.7% and 53.3% [3-4].

Select cases of adults with cortical TICAs after skull fracture, craniotomy, or external ventricular drain (EVD) placement have been described [6-9]. Here we present an unusual case of a patient who sustained massive blunt trauma and underwent an emergent surgery and later developed a delayed rupture of a cortical TICA.

## Case presentation

**Figure 1:**
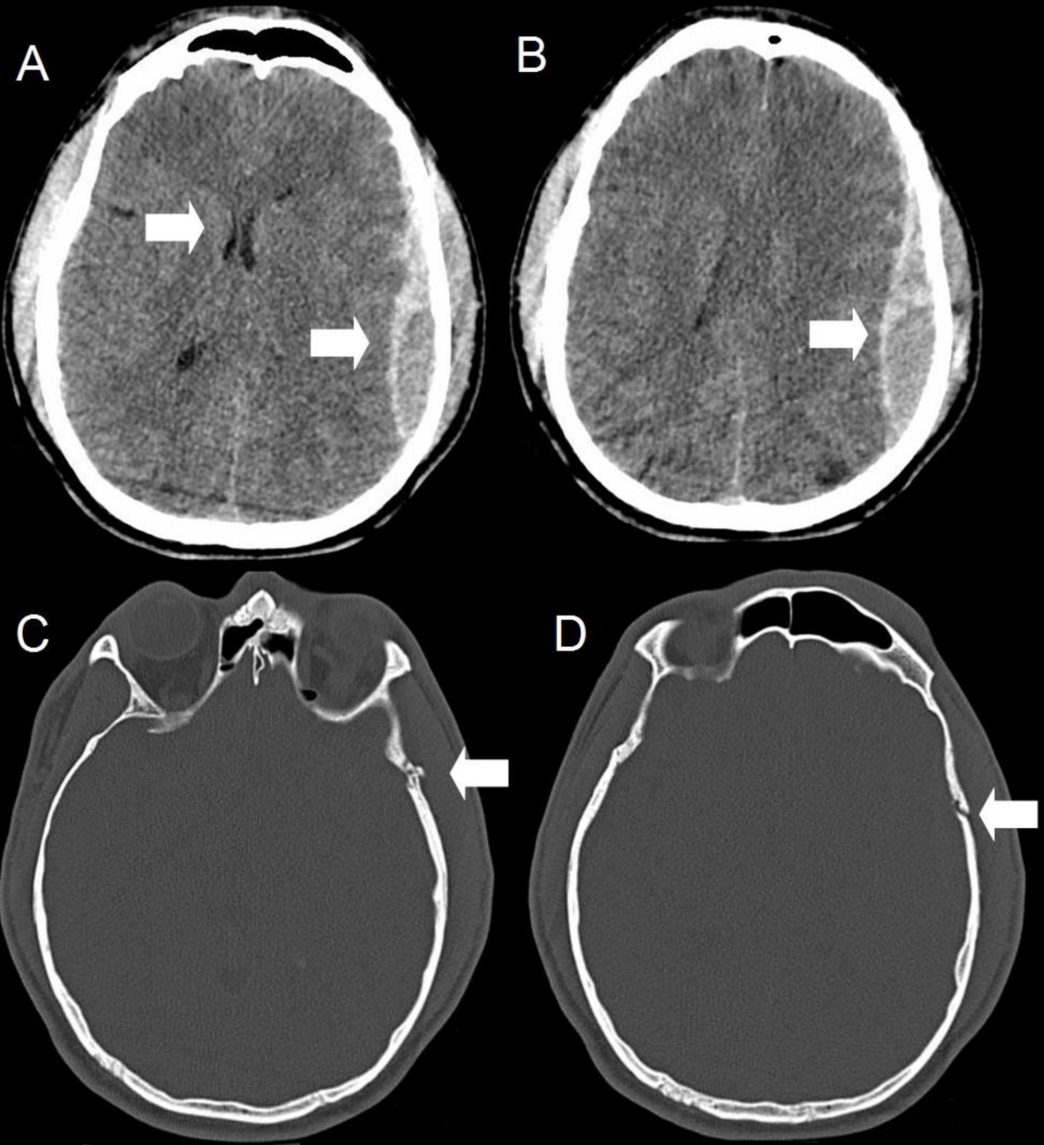
Computed tomography upon initial presentation after blunt trauma Axial (A, B, C, and D) slices show an acute left frontoparietal epidural hematoma of 19-mm thickness with a 5-mm midline shift. There is also a comminuted, non-depressed left temporal bone fracture.

A 29-year-old male with no significant past history presented with a Glasgow Coma Score (GCS) of 10 after falling out of a three-storeyed building onto his head. Imaging showed a 19-mm-thick left epidural hematoma with a 5-mm midline shift, as well as a comminuted left temporal bone fracture (Figure 1).

He was taken emergently to the operating room. Given the significant mechanism, he was presumed to be at a high risk of cerebral edema and therefore underwent a decompressive left hemicraniectomy, duraplasty, and placement of a left frontal EVD. Intra-operatively, a small subdural hematoma (SDH) was found originating from the cortical veins near the sylvian fissure. Hemostasis was achieved using bipolar cautery and Surgicel® (Johnson & Johnson, New Brunswick, NJ, USA). No aneurysms or unusual bleeding were noted. Immediate postoperative computed tomography showed a reduced mass effect with no atypical residual bleeding. He was discharged on postoperative day (POD) 17 to an in-patient rehabilitation unit with a GOS 3. Eventually, he was able to return home functionally independent and without neurologic deficits.

**Figure 2:**
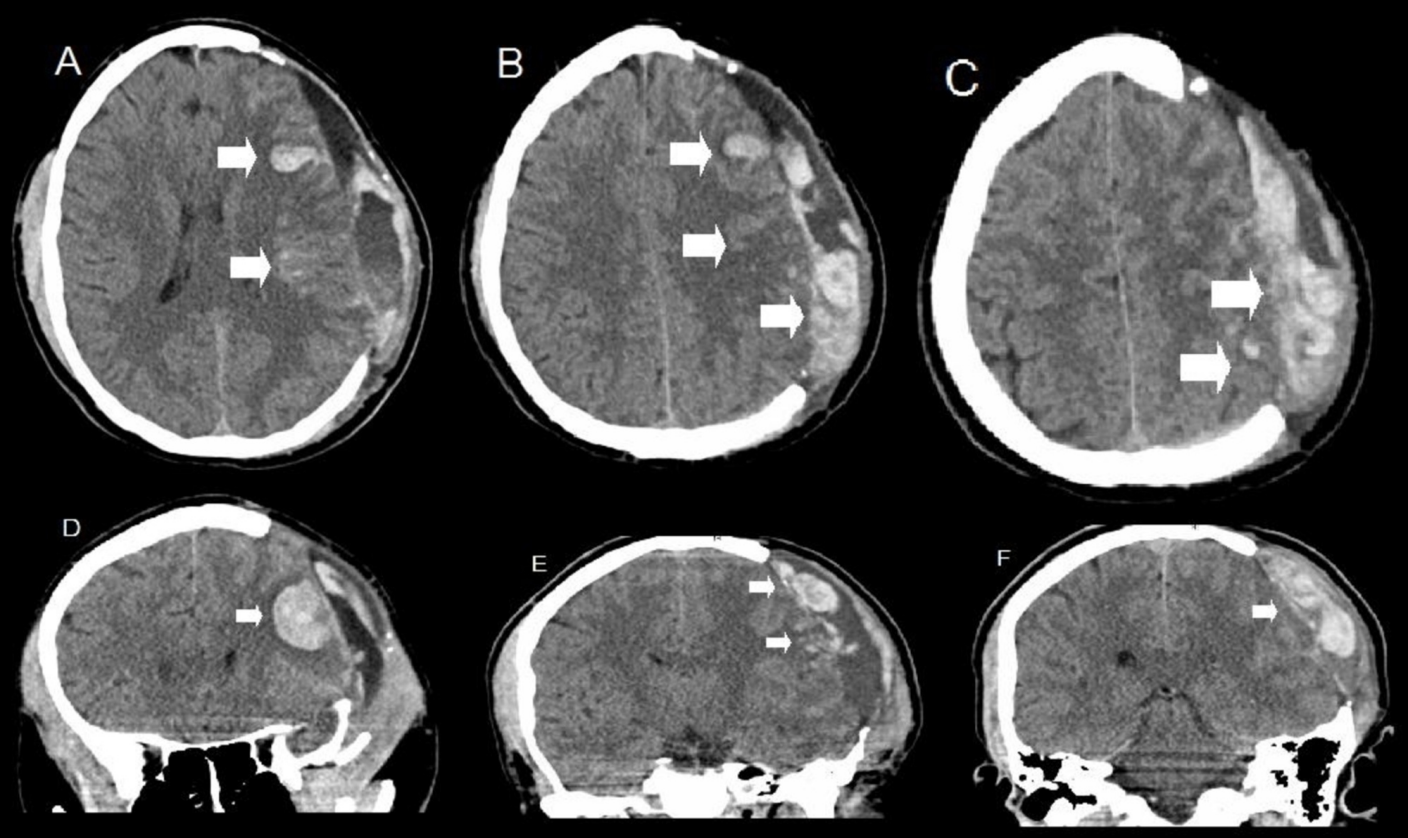
Computed tomography upon re-presentation three months after blunt trauma and craniectomy Axial (A, B, and C) and coronal (D, E, F) slices show scattered subarachnoid hemorrhage and mixed-density subdural blood products in the left parietal region as well as a singular parenchymal hematoma in the left frontal lobe. This bleeding pattern was unusual given absent known mechanism. There are post-surgical changes from a craniectomy on previous admission.

Three months postoperatively, he presented after being found on the ground unresponsive at home. He had a GCS 9, and there were no external signs of trauma. Imaging showed an unusual pattern of subarachnoid hemorrhage and SDH in the left frontoparietal region without mass effect (Figure 2).

**Figure 3:**
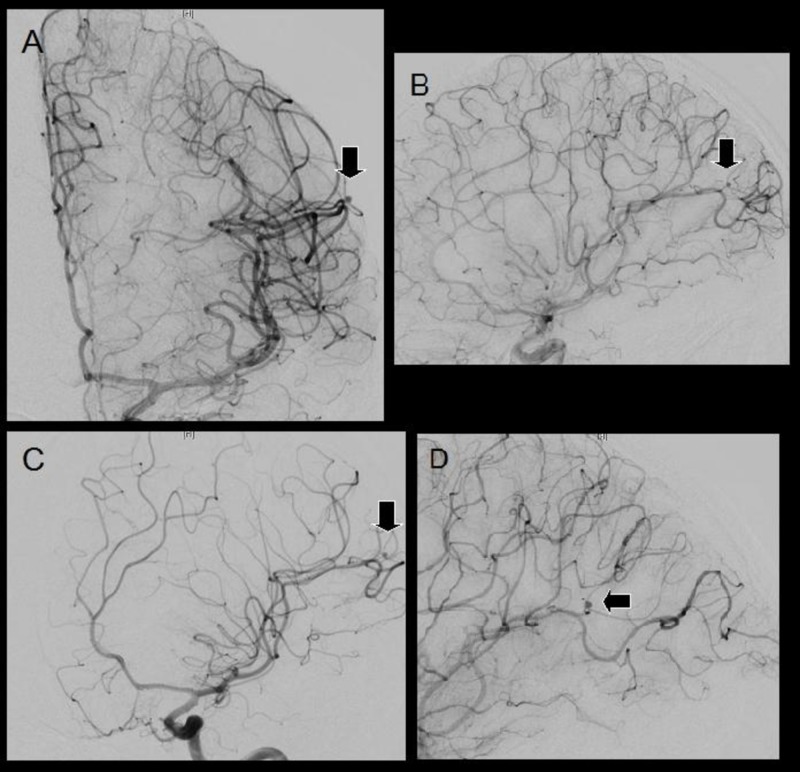
Digital subtraction angiography after craniectomy for trauma Anterior-posterior (A), lateral (B), and oblique (C and D) views showing a 2 x 2-mm distal left middle cerebral artery saccular traumatic intracranial aneurysm. The aneurysm is cortical and not located at a typical branching point and has a poorly defined neck and delayed emptying.

Given the uncertain history and atypical imaging, the patient underwent a four-vessel digital subtraction angiogram (DSA). Imaging showed distal left middle cerebral artery aneurysm (Figure 3).

This discovery substantially changed management. He promptly underwent a left craniotomy for aneurysm clipping. Stealth-guided imaging was used in designing the craniotomy. Intra-operatively, the dome appeared grossly composed of adventitia. The neck was dissected and a single clip was placed. Postprocedure angiogram demonstrated no residual filling. His recovery was uneventful, and he was discharged home on POD nine at his neurologic baseline with a GOS 5.

## Discussion

Diagnosis of TICAs can be practically challenging. Some have proposed screening DSA for fractures in the carotid canal and those with penetrating injury entering the pterional region, going through the middle cerebral artery territory, and crossing the midline [10]. We would not recommend screening DSA simply for significant blunt trauma with temporal bone fractures, even after this case report. It should be noted that TICAs may be obscured by post-surgical changes or may be occult and enlarge over time. Ultimately, any unusual bleeding pattern should be investigated: either by computed tomography angiography or as we prefer, a more sensitive DSA. However, the timing and the number of follow-up studies are highly controversial. This case report iterates the importance of understanding rare disease entities that occasionally present in a delayed fashion, and most importantly, being able to recognize atypical bleeding patterns and subtle angiographic findings that can dramatically change patient care. 

Management suggestions for TICAs are primarily inferred from a small case series [2-5,10]. Both open and closed treatment modalities have reported favorable results [3,10]. Aneurysm location, collateral circulation, and patient clinical condition dictate individualized decisions about treatment timing and technique. Morphology warrants special consideration since saccular TICAs have a greater risk of rupture compared to fusiform [5]. Endovascular techniques offer advantages including simultaneous diagnosis, shortened anesthesia, and reduced manipulation of the adjacent structures. Conversely, operative techniques offer advantages including definitive isolation, option to reconstruct the parent vessel, and relief of mass effect.

In our opinion, TICAs represent dangerous lesions that warrant prompt treatment upon discovery. Stealth-guided imaging is helpful to locate cortical aneurysms where precise exposure is important both in limiting manipulation and visualizing parent vessels. Delicate dissection through a scar or an edematous traumatic tissue is difficult and is done patiently with micro-instrumentation. TICA walls are extremely fragile, and the surgeon must be prepared for intra-operative rupture. Traditional clipping may not always be feasible if an aneurysm is deemed too delicate, has ruptured, or the neck is poorly defined. Alternatives include trapping, wrapping, or excision with or without parent vessel reconstruction. During the closure, any bleeding should be controlled with meticulous hemostasis under direct visualization.

The precise etiology, natural history, and diagnostic histology, in this case, are unknown. No dedicated vascular imaging studies were obtained during the initial hospitalization, given the typical presentation, blunt injury, and the lack of unusual bleeding on pre- and post-op imaging as well as intra-operatively. Therefore, we cannot definitively state if the cause of the TICA was due to the initial significant blunt trauma, iatrogenic, or delayed friction from the craniectomy bone edges or protective helmet; and secondly, we cannot comment on the time of origin, development, and rupture. The lack of outward trauma with no history on re-presentation three months later made us most suspicious of spontaneous TICA rupture as the cause of bleeding; although bleeding from an unknown trauma, given his status post craniectomy could not be entirely ruled out. Lastly, after clipping, the aneurysm dome was not extirpated and sent for diagnostic histology due to lack of clinical benefit and unacceptable risk. However, pre-operative DSA was most consistent with a TICA. Thus, in our opinion, the most likely diagnosis and the subsequent management of a ruptured TICA was made based on clinical history without an obvious mechanism, bleeding pattern, and angiographic features.

## Conclusions

In conclusion, we present an uncommon case of a delayed rupture of a cortical TICA. The natural history of these lesions is unknown, and developing a comprehensive screening protocol is difficult. Clinicians should consider this disease entity with an unusual history or bleeding pattern since angiographic findings can dramatically change decision making. Treatment plans should be individualized and performed urgently. Craniotomy for clipping of a cortical TICA is feasible, even in post-surgical patients.
